# mRNA-Mediated Duplexes Play Dual Roles in the Regulation of Bidirectional Ribosomal Frameshifting

**DOI:** 10.3390/ijms19123867

**Published:** 2018-12-04

**Authors:** Wan-Ping Huang, Che-Pei Cho, Kung-Yao Chang

**Affiliations:** Institute of Biochemistry, National Chung-Hsing University, 145 Xingda Road, Taichung 402, Taiwan; f32114702@gmail.com (W.-P.H.); andycho1217@gmail.com (C.-P.C.)

**Keywords:** mRNA-ribosome interplay, hungry codon, programmed ribosomal frameshifting, Shine-Dalgarno sequence

## Abstract

In contrast to −1 programmed ribosomal frameshifting (PRF) stimulation by an RNA pseudoknot downstream of frameshifting sites, a refolding upstream RNA hairpin juxtaposing the frameshifting sites attenuates −1 PRF in human cells and stimulates +1 frameshifting in yeast. This eukaryotic functional mimicry of the internal Shine-Dalgarno (SD) sequence-mediated duplex was confirmed directly in the 70S translation system, indicating that both frameshifting regulation activities of upstream hairpin are conserved between 70S and 80S ribosomes. Unexpectedly, a downstream pseudoknot also possessed two opposing hungry codon-mediated frameshifting regulation activities: attenuation of +1 frameshifting and stimulation of a non-canonical −1 frameshifting within the +1 frameshift-prone CUUUGA frameshifting site in the absence of release factor 2 (RF2) in vitro. However, the −1 frameshifting activity of the downstream pseudoknot is not coupled with its +1 frameshifting attenuation ability. Similarly, the +1 frameshifting activity of the upstream hairpin is not required for its −1 frameshifting attenuation function Thus, each of the mRNA duplexes flanking the two ends of a ribosomal mRNA-binding channel possesses two functions in bi-directional ribosomal frameshifting regulation: frameshifting stimulation and counteracting the frameshifting activity of each other.

## 1. Introduction

Genetic codes and potential secondary structures defined by local RNA sequences constitute the two layers of information embedded within the primary mRNA sequences of an open reading-frame (ORF). The secondary structures in mRNA are unwound by an intrinsic duplex-unwinding activity of ribosome during translation [1,2], allowing exposure of the buried genetic codes for decoding in the ribosomal A-site, while refolding co-translationally after leaving the ribosome. As an mRNA is translated by multiple ribosomes, the unwinding and refolding cycles of secondary structures repeat for an active mRNA under translation. However, specific mRNA structures could resist ribosomal unwinding and trigger backward slippage (toward the 5’-direction of mRNA) of the ribosome by one nucleotide within a slippery sequence to stimulate translational reading-frame switch toward the −1 frame. An in-frame XXXYYYZ slippery sequence as well as an optimally placed (5–8 nucleotides) downstream stimulator pseudoknot [3] or hairpin [4] is required for efficient −1 programmed ribosomal frameshifting (PRF) with X and Y each representing an identical nucleotide. By positioning A- and P-site tRNAs over the slippery sequence while keeping Watson-Crick base-pairing identities of the codon-anticodon interactions at the first two positions of each codon unchanged in 0- and −1 frames, this configuration helps increase probability to slip the ribosome one nucleotide in the 5’ direction [5,6]. By contrast, specific mRNA signals can program a ribosome to slip forward (toward the 3’-direction) by a single nucleotide for +1 frameshifting [7,8,9]. The well-documented +1 frameshifting in the Ty1 retrotransposon in *Saccharomyces cerevisiae* involves a hepta-nucleotide sequence (CUUAGGC), and is caused by ribosome pausing at the AGG codon due to low expression levels of AGG decoding tRNA while being decoded within ribosomal A-site [10]. Differences in *cis*-element requirement between +1 and −1 frameshifting suggest that distinct mechanisms are responsible for these two frameshifting pathways, although disruption of codon-anticodon interactions between peptidyl-tRNA and mRNA during translocation step of translation elongation has been proposed to play an initiation role for frameshifting [11].

Both +1 and −1 PRFs have been characterized in prokaryotic gene expression regulation [7,8,9]. +1 PRF is adopted by the *prfB* gene of *E. coli.* to encode one of the translation termination proteins, release factor 2 (RF2), in a regulatory feedback loop. When the UGA recognizing RF2 is insufficient in a cell, a translating ribosome pauses at the stop codon within the CUUUGA sequence located in the N-terminal coding region of *prfB*. To escape from this trap, the paused ribosome slips one nucleotide forward to continue translation in +1 frame to complete the synthesis of full-length RF2. This results in supply of mature RF2 to terminate translation at the frameshifting site UGA codon to prevent synthesis of more RF2. By contrast, the *dnaX* gene of *E. coli.* produces the γ subunit of DNA polymerase III by −1 PRF via an AAAAAAG slippery sequence (A6G) and a downstream RNA hairpin. [12]. Single-molecule fluorescence analysis tracking dye-labeled ribosome movement along mRNA suggested that the downstream dnaX hairpin impeded translocation while stimulating −1 frameshifting at AAG codon during the incoming accommodation step [13]. However, a kinetic analysis of a downstream −1 PRF pseudoknot in an in vitro 70S translation system argued that the frameshifting event occurred in the post-translocation step after peptide bond formation between the aminoacyl-tRNA and peptidyl-tRNA that sit at the XXY and YYZ codons, respectively [14]. Nevertheless, these works help establishing 70S ribosome as an ideal platform for mechanical analysis of frameshifting regulation.

In addition to specific downstream structures, an internal Shine-Dalgarno sequence (SD) 8 to 11 nucleotides upstream of the slippery site was shown to act as a −1 PRF stimulator [12]. Furthermore, E-site juxtaposing internal SD upstream of the CUUUGA +1 frameshifting site can promote +1 frameshifting [15], whereas deletion analysis indicated that E-site juxtaposing upstream internal SD can downregulate −1 PRF stimulated by a downstream dnaX hairpin [12]. Although internal SD ● anti-SD interaction has been suggested to be an elongation pausing element for 70S ribosome [16], mechanisms underlining the opposing effects in +1 and −1 frameshifting regulation by E-site juxtaposing upstream internal SD remain unclear. Recently, it was found that a potential RNA hairpin upstream of XXXYYYZ −1 frameshifting site can down-regulate mammalian −1 PRF as well as stimulate AGG codon mediated +1 frameshifting in *Saccharomyces cerevisiae* [17,18]. The frameshifting regulation activities were shown to be positively correlated with the predicted stability of the upstream hairpins. Given the unfolding/refolding cycles of a translating mRNA, it suggests the involvement of a co-translationally refolded upstream hairpin in frameshifting regulation when the ribosome moves into a downstream frameshifting site. These findings of unexpected frameshifting regulation by upstream structures are in contrast to the well-known role of downstream RNA structures in stimulation of eukaryotic −1 PRF [3,4] and implicate a link between the mechanisms of +1 and −1 frameshifting regulation by mRNA structures flanking frameshifting sites. However, differences of sequence compositions between the +1 and −1 frameshifting sites hamper further analysis. Intriguingly, a trans-formed duplex mediated by an antisense DNA and the sequence upstream of frameshifting site can replace the cis-acting hairpin to attenuate −1 PRF [19]. In addition to suggesting that formation of an upstream duplex juxtaposing frameshifting sequences is the determinant for frameshifting regulation, this result is reminiscent of the duplex formed between the internal SD sequence and 16S rRNA in prokaryotic frameshifting regulation [12,15] although the components forming the two duplexes are different. Therefore, a direct comparison of frameshifting regulation activities between upstream hairpins and internal SD mediated duplexes in 70S ribosome could shed light to help resolving the roles of mRNA structures flanking the ribosomal mRNA-binding channel in frameshifting regulation.

In this study, the frameshifting regulation activities of upstream refolding hairpins were examined using the well-characterized −1 frameshift-prone A6G and the +1 frameshift-prone CUUUGA frameshifting sites as the model systems. In addition to showing that a stable upstream hairpin is a functional mimicry of upstream internal SD in both +1 and −1 frameshifting regulations, we discovered a downstream structure-mediated non-canonical −1 frameshifting under the +1 frameshift-prone CUUUGA frameshifting site in the absence of RF2. Importantly, by using this frameshifting site to minimize the effects of sequence composition differences in P-site codon-anticodon interactions, we are able to demonstrate that a downstream structure can stimulate −1 frameshifting and attenuate +1 frameshifting, which are opposite to the frameshifting regulation activities of the upstream refolding hairpin. These findings indicate that stable structures flanking the two ends of mRNA-binding channel within an elongating ribosome play opposite roles in regulation of bidirectional reading-frame switch. In addition to providing a framework for further mechanism analysis, these results can be used to explain non-canonical frameshifting events observed in disease-related genes containing long expanded CAG trinucleotide repeat expansions.

## 2. Results

### 2.1. Distinct Upstream Duplexes Stimulate +1 Frameshifting and Attenuate −1 Frameshifting in an In Vitro 70S Translation System

Cell-free in vitro translation lysates of *E. coli.* have been used to reproduce cellular translation mediated processes, including ribosomal frameshifting for mechanism analysis [20,21]. To examine the frameshifting regulation activity of upstream refolding hairpins in 70S ribosome, a glutathione-S-transferase (GST) and *Renilla* luciferase fusion protein coding construct (GST-Rluc) was designed to serve as the in vitro frameshifting reporter. The GST open reading frame (ORF) was fixed in 0 frame, while the downstream Rluc ORF was fused out of frame such that it could only be accurately translated upon frameshifting through insertion of translational frameshifting signals between GST and Rluc ORFs (Figure S1A). Premature stop codons for out of frame translation were also inserted in the N-terminal region of Rluc ORF (Figure S1A). They were used to prevent radioactivity-based frameshifting efficiency calculation bias caused by ribosome drop-off effect [22] during translation of long frameshifted polypeptides. In vitro −1 and +1 PRF activities using different amounts of reporter mRNA as translation templates in cell-free lysates of *E. coli.* (Figures S1B,C and S2A) were examined to determine the optimal amount of mRNA (200 ng) required for frameshifting assay. The −1 PRF efficiencies induced by a downstream dnaX hairpin in the presence of upstream internal SD mediated duplexes with different spacings toward the frameshifting site (Figure S1B,D) were similar to those reported in vivo [12], indicating that the experimental platform can faithfully reproduce this −1 PRF model system.

**Figure 1:**
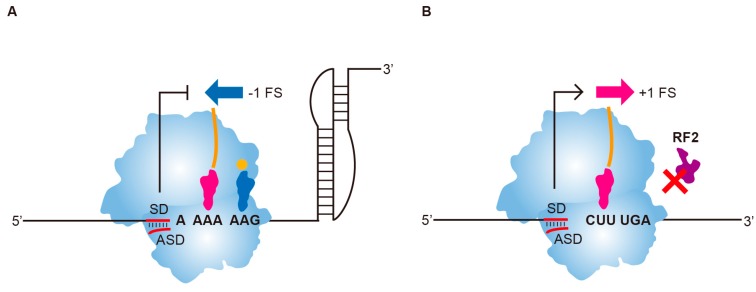
Frameshifting regulation activity comparison of upstream internal SD-mediated duplexes in −1 and +1 frameshifting model systems of *E. coli*. (**A**) The −1 frameshifting attenuation activity of internal SD-mediated duplex on A6G −1 frameshifting site with a downstream pseudoknot. The blue arrow indicates stimulation of −1 FS by downstream structure, whereas T-bar indicates the attenuation of −1 FS by E-site juxtaposing internal SD. (**B**) The +1 frameshifting stimulation activity of internal SD-mediated duplex on CUUUGA +1 frameshifting site in the absence of RF2. The rose red arrow indicates stimulation of +1 FS by E-site juxtaposing internal SD.

**Figure 2:**
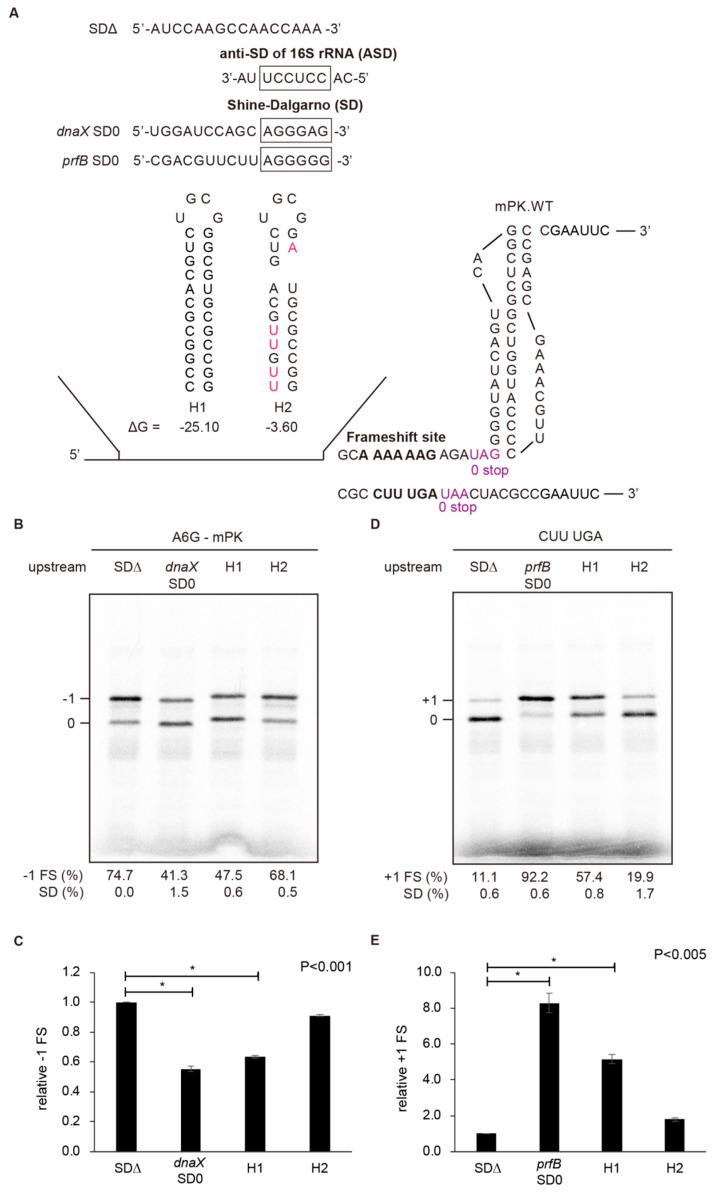
Stimulation of +1 frameshifting as well as attenuation of −1 frameshifting by internal SD-mediated duplex mimicries in an in vitro 70S translation system. (**A**) Sequences, predicted secondary structures and free energy of distinct upstream hairpins evaluated. Mfold [25] was used for mRNA secondary structure and free energy prediction. Effects of upstream duplex variants on −1 and +1 frameshifting were evaluated using respective A6G and CUUUGA frameshifting sites inserted in pET GST-Rluc-1 reporters. Due to the exclusion of RF2 in the reaction, a UAA or UAG stop codon was used as the 0 frame stop codon (in purple color), while UAA was used as the out of frame premature stop codons in the N-terminal region of Rluc. Mutations to disrupt the stem of H1 were typed in rose-red color. (**B**) Radioactivity-based −1 PRF assay by SDS-PAGE analysis of ^35^S methionine-labeled in vitro translation products, with calculated −1 PRF efficiencies shown, for different upstream duplex constructs in (**A**). The values displayed are means ± SD of three independent experiments. (**C**) Relative −1 PRF activity of frameshifting activities in (**B**) compared to SDΔ with asterisks indicating *p* value < 0.001 using Student’s *t*-test. (**D**) Radioactivity-based +1 PRF assay by SDS-PAGE analysis of ^35^S methionine-labeled in vitro translation products, with calculated +1 frameshifting efficiencies shown, for different upstream duplex constructs in (**A**). The values displayed are means ± SD of three independent experiments. (**E**) Relative +1 PRF activity of (**D**) compared to SDΔ with asterisks indicating *p* value < 0.005 using Student’s *t*-test.

To compare upstream hairpins with SD ● anti-SD duplexes in frameshifting regulation, we used the A6G slippery sequence with a modified pseudoknot (mPK) derived from the infectious bronchitis virus (IBV) *1a/1b* gene [14,23], and the CUUUGA frameshifting site of RF2 for −1 and +1 PRF analysis, respectively (Figure 1). A CGC sequence was used as the E-site codon for the CUUUGA frameshifting site to avoid E-site invasion [24] that could mask the +1 frameshifting stimulation activities of upstream duplexes. The effects of upstream internal SD-mediated duplexes and hairpins (Figure 2A) on −1 and +1 PRF efficiencies were analyzed. An RNA hairpin of a GC base-pair rich 12 base-pairs stem capped by UGCG loop sequence (H1) with a predicted stable hairpin conformation was used for comparison (Figure 2A). We found that the SD-ASD duplex attenuated activity of −1 PRF stimulated by mPK the most (Figure 2B,C), and was also the most potent stimulator of +1 PRF for the CUUUGA frameshifting site in the absence of RF2 (Figure 2D,E). By contrast, RNA hairpins regulated +1 and −1 frameshifting efficiencies to a lesser extent. However, the stable upstream hairpin, H1 reduced −1 PRF to a similar extent as that of internal SD and possessed about 60% of the +1 frameshifting activity that can be stimulated by the internal SD (Figure 2D,E). By contrast, a destabilization mutant of H1 with reduced stability through Watson-Crick base-pairing disruption and bulge insertion (H2) possessed much lower −1 PRF attenuation and +1 PRF stimulation activities compared with those of H1. These results are consistent with the positive correlation between frameshifting regulation activity and hairpin stability observed before [18]. Furthermore, the upstream hairpin-dependent +1 frameshifting occurred only in the absence of RF2 (Figure S2B). Together with previous analysis in eukaryotic systems [19], these results indicate that stable upstream refolding hairpins are functional elements capable of regulating frameshifting in both 70S and 80S ribosomes.

### 2.2. A Downstream mPK Plays Opposite Roles in +1 and −1 Frameshifting Regulation

**Figure 3:**
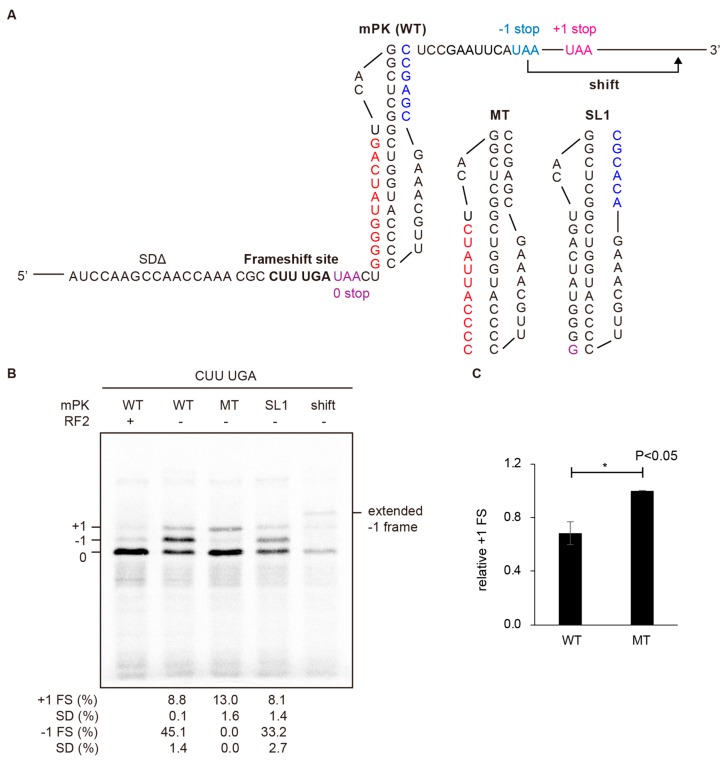
Reduced +1 frameshifting by a downstream pseudoknot coexists with an unusual frameshifting event in the absence of RF2. (**A**) Sequences of the constructs designed to evaluate +1 attenuation activity of downstream mPK pseudoknot variants. MT and SL1 are the variants of mPK with mutations (in colors) to destabilize the stem1 and stem2, respectively. Please note that there are three UAA stop codons (with different colors) to terminate translation in three different frames in the absence of RF2 in these constructs using the pET GST-Rluc-1 reporter (Figure S1A) as the backbone. The −1 and +1 frame UAA stop codons are located in the N-terminal region of Rluc ORF with the −1 frame stop codon being moved to downstream of +1 frame stop codon in the “shift” construct. (**B**) The +1 frameshifting attenuation assays by SDS-PAGE analysis of ^35^S methionine-labeled in vitro translation products for different mPK variant constructs in (**A**), with +1 PRF and potential −1 PRF products annotated. Calculated −1 and +1 PRF efficiency values displayed are means ± SD of three independent experiments. (**C**) Relative +1 PRF activity of construct with downstream WT mPK compared to MT with asterisks indicating *p* values < 0.05 using Student’s *t*-test.

The ability to stimulate +1 PRF and attenuate −1 PRF by a stable upstream hairpin implies a link between +1 frameshifting stimulation and −1 frameshifting attenuation. It also raises the question of whether a downstream −1 PRF stimulator also plays an opposite role in −1 and +1 frameshifting regulation. This means that a downstream −1 PRF stimulator may serve as a +1 PRF attenuator. To test this hypothesis, mPK was placed downstream of the +1 frameshift-prone CUUUGA sequence (Figure 3A) to examine its effect on +1 frameshifting in the absence of RF2. A mutant of mPK with stem1 disrupted (MT) was used as the negative control for measuring intact +1 frameshifting activity. Interestingly, +1 frameshifting activity decreased when the downstream structure was replaced from MT to mPK (Figure 3B,C). This result is consistent with +1 frameshifting attenuation by a downstream mPK. Thus, the mPK downstream of mRNA-binding channel of an elongating ribosome plays reverse regulatory roles in +1 and −1 frameshifting regulations.

### 2.3. An Unusual −1 Frameshifting Event is Triggered in a +1 Frameshift-Prone Sequence by Downstream Structures in the Absence of RF2

Unexpectedly, an extra translation product appeared when the CUUUGA sequence was followed by an mPK or a hairpin in the absence of RF2 (Figure 3B lanes 2 and 4), whereas it disappeared when RF2 was added (lane 1) or the downstream mPK was replaced with an MT (lane 3). Sequence analysis implicated that the extra translation product could arise from a −1 frameshifting event although there was no XXXYYYZ consensus slippery sequence of −1 PRF. Consistently, mutating the potential −1 frame stop codon moved the extra translation product to a higher region of the gel, corresponding to translation termination at the next −1 frame stop codon (“shift” in Figure 3B). Together, these results suggest that a non-canonical −1 frameshifting may have occurred in addition to +1 frameshifting. Finally, faint extra bands with low mobility in the gel appeared upon longer image exposure and disappeared upon treatment of the translation reaction with RNase before gel-loading (Figure S3). Given that the translated protein products were ^35^S-methionine labelled, the senstivity to RNase treatment suggested that the low mobility bands belonged to colvant linkage adducts between proteins and RNAs, and most probably a peptidyl-tRNA intermediate during translation in the absence of RF2.

**Figure 4:**
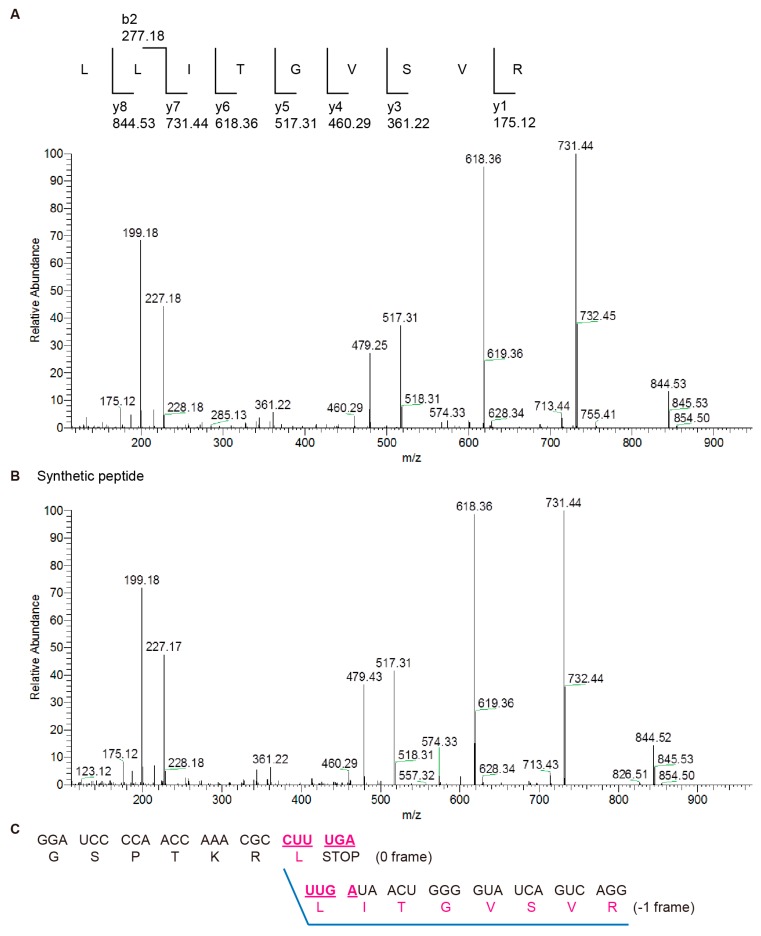
Non-canonical −1 frameshifting confirmed by mass spectrometry analysis. (**A**) Fragmentation spectrum of the frameshifting site spanning peptide recovered from in-gel digestion sample. The fragment ions identified are indicated on top. (**B**) Fragmentation spectrum of chemically synthesized frameshifting site spanning peptide standard. (**C**) Nucleotide sequences spanning the 0 frame/−1 frame junction and corresponding amino acids encoded in the two different frames with the characterized LLITGVSVR peptide sequences underlined in blue color.

To confirm the identity of the extra translation product, a FLAG-tagged construct (FLAG-CUUUGA-mPK) (Figure S4A) was designed to help enrich the recovery of potential −1 frame products for mass spectrometry analysis. The nucleotide sequence encoding the FLAG amino acid sequence was inserted upstream of the corresponding frameshifting site in the 0 frame of GST-Rluc fusion ORF so that translation products, including shifted and non-shifted ones all contain the FLAG fragment. After translation, these FLAG-tagged translation products were immuno-precipitated by anti-FLAG antibody, purified by protein G conjugated bead, and analyzed by SDS-PAGE (Figure S4B–E). Comparison of the frameshifting products of the construct before and after immunoprecipitation with those of the FLAG-less frameshifting reporter indicated that frameshifting efficiency remained similar between the two constructs (Figure 3 and Figure S4D). Attempts of LC-MS/MS analysis by thrombin treatment to remove GST from the −1 frameshifted C-terminal peptide were not successful (data not shown). Instead, in-gel trypsin digestion was performed to generate peptide fragments for LC-MS/MS characterization. Analysis of liquid chromatography tandem MS (LC-MS/MS) data of peptide fragments derived from the unusual translation product identified a 9 amino acid tryptic peptide fragment (LLITGVSVR) and this was further validated using a chemically synthesized peptide standard (Figure 4A,B). This result supports a frameshifting event occurring at the CUUUGA sequence with a UGA to UUG shift (encoding the second leucine after formation of the first leucine by decoding the 0 frame CUU), followed by continued translation extension in the −1 frame (Figure 4C and Figure S4F).

### 2.4. Efficient Non-Canonical −1 Frameshifting Requires an Identical Nucleotide Bridging E- and P-Sites in Addition to a Downstream Structure

**Figure 5:**
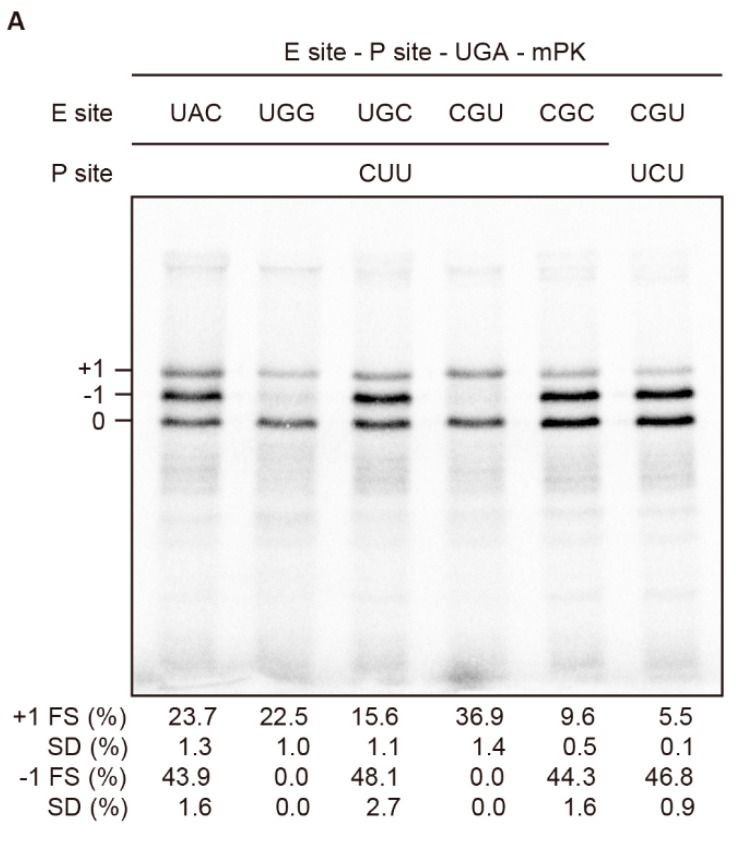
An identical nucleotide bridging E- and P- sites facilitates non-canonical −1 frameshifting. SDS-PAGE analysis of in vitro radioactivity based frameshifting assays of constructs with E-site or P-site mutagenesis. Calculated −1 and +1 PRF efficiencies displayed are means ± SD of three independent experiments.

As the first U of the E-site UAU codon juxtaposing the CUUUGA +1 frameshifting site in wild-type RF2 was reported to involve E-site invasion through base-pairing with anti-SD in 16S rRNA [24], we performed E-site sequence mutagenesis to explore the mechanism of non-canonical −1 frameshifting. Interestingly, replacing E-site sequence from CGC to CGU dramatically diminished the −1 frameshifting product with the restoration of +1 frame product (compare lanes 4 and 5 in Figure 5A), whereas replacement from CGC to UGC did not lead to a diminished −1 frameshifting product (compare lanes 3 and 5 in Figure 5A). Further mutating UGC to UAC to reduce codon-anticodon stability did not alter the distribution of frameshifting patterns (lanes 1 and 3 in Figure 5A), whereas mutating UGC to UGG almost abolished the −1 frameshifting product (lane 2 in Figure 5A). Thus, neither stability of E-site codon-anticodon pairing nor E site invasion is the main cause of the dramatic switch in frameshifting pathways. Although the diminished −1 frameshifting can be explained by −1 PRF efficiency modulation by E-site sequence composition [26,27], we noticed that significant −1 frameshifting seems to require the third position of the E-site codon to be a C nucleotide, which also occurs at the first position of the P-site codon. Furthermore, dramatic −1 frameshifting reduction by converting CGC CUU to CGU CUU can be reversed by further mutating the P-site sequence from CUU to UCU (lanes 4–6 in Figure 5A), resulting in an identical U bridging the E- and P- sites. Together, these data indicate that an identical nucleotide in the two positions bridging 0 frame E- and P- sites is enough to facilitate −1 frameshifting with a downstream structure in the absence of RF2, and is consistent with the repairing of 0 frame peptidyl-tRNA to an identical nucleotide in the first position of the −1 frame codon to pave the way for −1 frame switching.

**Figure 6:**
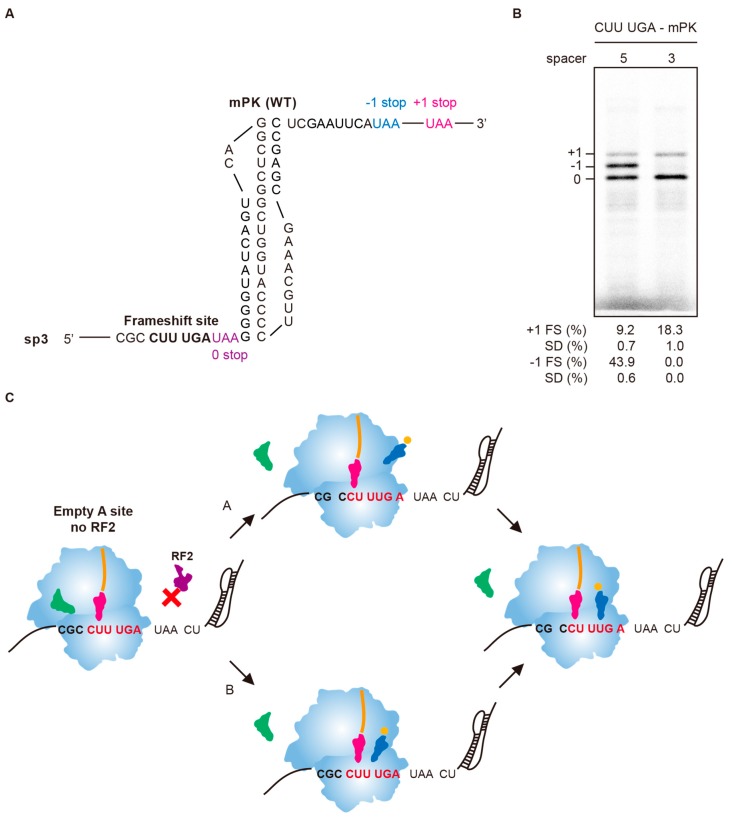
The downstream mPK stimulates −1 frameshifting with a five-nucleotide spacing toward the A-site sitting UGA codon. (**A**) The sequence and predicted structure of sp3 construct. (**B**) Radioactivity-based frameshifting assays of the sp3 construct (corresponding to spacer 3 model) compared with the CUUUGA-mPK construct (corresponding to spacer 5 model) in Figure 3A. Calculated −1 and +1 PRF efficiency values displayed are means ± SD of three independent experiments. (**C**) Proposed mechanisms of the non-canonical −1 PRF in the absence of RF2. The proposed mechanisms put the UGA stop codon in the A-site, resulting in a spacer of 5 nts to mPK while the CUU codon is sitting in the P-site based on the data from (**B**) as well as the requirement of RF2 deficiency for the non-canonical −1 frameshifting. This positioning allows the downstream mPK to stimulate −1 frameshifting through two alternative pathways with differences in the sequential order of peptidyl-tRNA movement from 0-frame to −1 frame. In pathway A, the 0-frame peptidyl-tRNA moves from CUU to −1 frame CCU and thus positions the UUG codon in the A-site. In pathway B, an incoming charged tRNA samples the available reading-frames while peptidyl-tRNA is sitting in the 0-frame P-site and is paved by two identical nucleotides bridging E- and P-sites that favors −1 frame accommodation of peptidyl-tRNA to facilitate −1 frameshifting.

mPK has been suggested to stimulate −1 PRF in a UUUAAAG slippery site during post-translocation after peptide bond formation while 0 frame UUA and AAG codons occupy ribosomal P- and A- sites, respectively [14]. Given that the CUU codon in the CUUUGA frameshifting site represents the last coding amino acid in the 0 frame, positioning the CUU codon into the A-site for formation of the final peptide bond results in a spacing of 8 nucleotides in length toward downstream mPK (Figure 3A). However, the absence of RF2 being required for frameshifting to occur indicates that frameshifting occurs after the UGA codon enters the ribosomal A-site, resulting in a spacing of 5 nucleotides in length toward mPK. We reasoned that if the first model can explain observed −1 frameshifting, deleting the spacer length by 2 nucleotides should still be −1 frameshifting competent (Figure 6A). By contrast, the second model predicted such a deletion mutant should be frameshifting incompetent because spacing toward the pseudoknot becomes non-optimal (3 nucleotides in length). The results showed that the resulted construct sp3 lost −1 frameshifting activity (Figure 6B), indicating that mPK stimulates −1 frameshifting with a five-nucleotides spacing downstream of the ribosomal A- site sitting UGA stop codon. Combined with −1 frameshifting product characterization and E-site sequence mutagenesis results, they suggest that the non-canonical −1 frameshifting occurs via a single slippage of peptidyl-tRNA in 5’ direction through two possible pathways with UGA stop codon sitting at the ribosomal A-site (Figure 6C). In pathway A, the 0-frame peptidyl-tRNA moves from CUU to −1 frame CCU, allowing the −1 frame UUG codon for decoding in the A-site. By contrast, an incoming UUG decoding charged tRNA could sample the available reading-frames in the empty A-site and is paved by the two identical nucleotides bridging E- and P-sites to accept the movement of peptidyl-tRNA toward the −1 frame, and resulting in −1 frame decoding in the A-site.

### 2.5. Stable mRNA Structures Flanking the mRNA-Binding Channel of an Elongation Ribosome Counteract Each Other’s Frameshifting Activity

**Figure 7:**
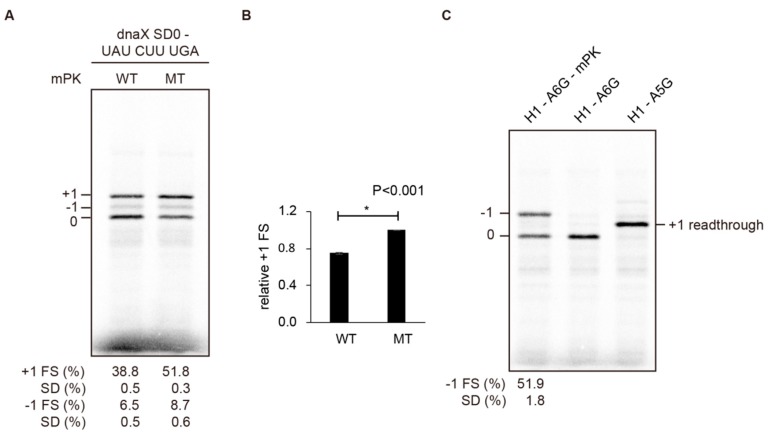
The −1 frameshifting is not required for +1 frameshifting attenuation activity of mPK. (**A**) SDS-PAGE analysis of in vitro radioactivity-based frameshifting assay of constructs containing CUUUGA frameshifting site with an UAU codon in the E-site, an upstream internal SD of dnaX (SD0), and different downstream mPK variants (WT and MT). The values displayed are means ± SD of three independent experiments. (**B**) Relative +1 PRF activity of (**B**) with asterisks indicating *p* value < 0.001 using Student’s *t*-test. (**C**) SDS-PAGE analysis of in vitro radioactivity based frameshifting assay of a construct with an A6G frameshifting site and a stable upstream H1 hairpin. A5G is a read-through control with the A6G slippery sequence mutated. The values displayed are means ± SD of three independent experiments.

That non-canonical −1 frameshifting occurred within a +1 frameshift-prone CUUUGA site with the presence of a downstream pseudoknot raises the possibility that reduced +1 frameshifting shown in Figure 3B is not caused by a direct counteracting downstream structure (an attenuator), but rather is the result of multiple frameshifting pathways competing. However, +1 frameshifting efficiency was reduced significantly by the downstream mPK (from 51.8% to 38.8%) in a UAUCUUUGA construct that favors +1 frameshifting but disfavors −1 frameshifting (Figure 7A,B). Therefore, the main cause of the observed +1 frameshifting reductions is not −1 frameshifting pathway competition, indicating direct counteraction to +1 frameshifting by the downstream mPK. To see if this property is also applicable to −1 frameshifting attenuation by an upstream hairpin, we constructed an H1 hairpin upstream of the A6G −1 frameshifting site. We did not detect +1 frameshifting activity (lane 2 in Figure 7C) although H1 did attenuate −1 PRF efficiently (lane 1 in Figure 7C). Taken together, these results indicate that the existence of a stable mRNA hairpin at either end of the mRNA-binding channel of an elongation ribosome counteracts frameshifting stimulated by its counterpart at the other end of the channel.

### 2.6. Co-Existence of +1 and −1 Frameshifting by Modulating the Stabilities of Structures Flanking the mRNA-Binding Channel of an Elongation Ribosome

**Figure 8:**
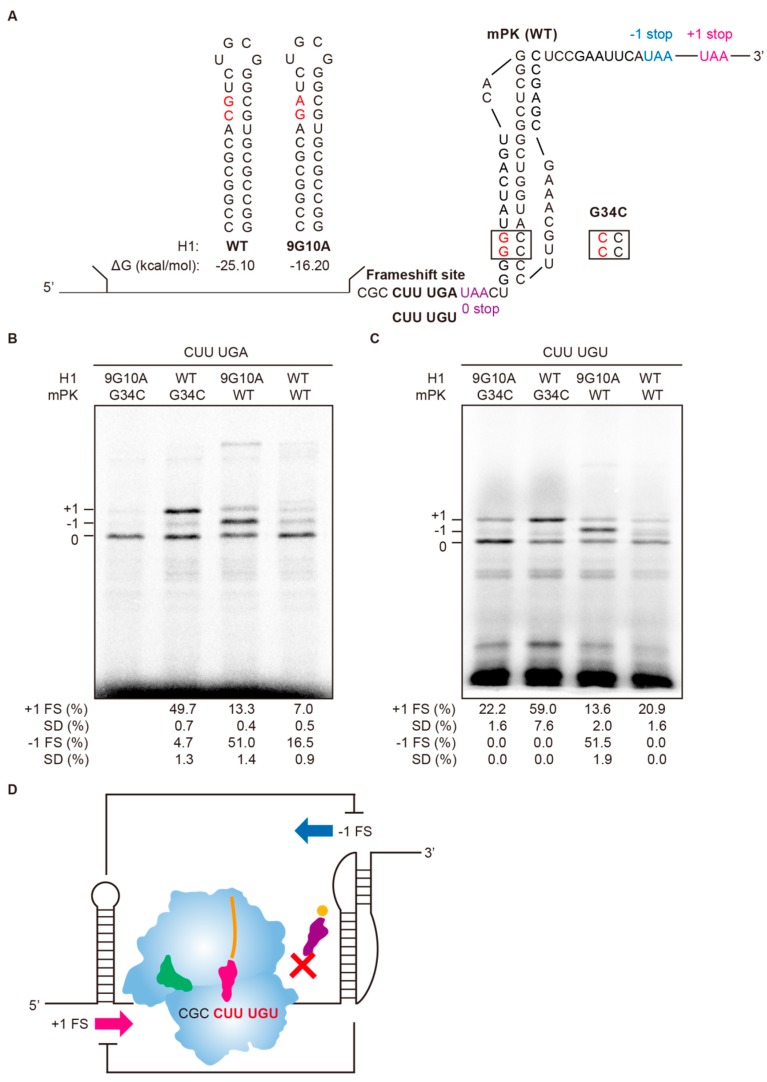
Bidirectional reading-frame switch regulation by structures flanking an empty A-site ribosome. (**A**) Partial sequences for reporter constructs inserted with CUUUGA frameshifting site and flanking structures of different stabilities. The A-site codon is UGA for RF2 exclusion frameshifting assay and is followed by a UAA 0-frame stop codon (colored in purple) to facilitate proper translation termination. The −1 and +1 frame UAA stop codons (in colors) are located in the N-terminal region of Rluc ORF. Mutations to destabilize the upstream or downstream structures are shown in red. (**B**) SDS-PAGE analysis of radioactivity-based frameshifting assay using constructs in (**A**) in the absence of RF2. (**C**) SDS-PAGE analysis of in vitro radioactivity-based frameshifting assay using constructs with CUUUGU frameshifting site and different flanking structures compared with those in (**A**). The experiments were performed under conditions with UGU as a hungry codon (Figure S6). Calculated −1 and +1 PRF efficiencies displayed are means ± SD of three independent experiments. (**D**) Scheme showing the opposite roles of flanking structures in bidirectional frameshifting regulation for an empty A-site ribosome.

To further explore the role of flanking structures in bidirectional frameshifting regulation, destabilizing mutations to destroy Watson-Crick base-pairing for stability reduction were introduced at the upper stem of upstream H1 hairpin (9G10A) and the stem 1 of downstream mPK pseudoknot (G34C) (Figure 8A) to examine the effects on +1 and −1 frameshifting in the absence of RF2. The CUUUGA sequence was weakly frameshifting-prone without the presence of a stable flanking structure (with the destabilized 9G10A and G34C mutants) (lane 1 in Figure 8B), while +1 frameshifting activity increased in the presence of a stable upstream hairpin (H1-WT) (lane 2). By contrast, a downstream mPK strongly stimulated −1 frameshifting (lane 3). Similar to that of Figure S3, an extra faint RNase-sensitive band with low mobility appeared but disappeared upon RNase treatment (Figure S5). The co-existence of this RNase-sensitive band with the −1 frameshifting product and its disappearance in the presence of RF2 implicated that it is related to −1 frameshifting and its identity awaits further characterization in the future. Finally, both +1 and −1 frameshifting products were significantly reduced in the presence of both the stable upstream H1 hairpin and the downstream mPK (lane 4). This result is consistent with mutual attenuation of +1 and −1 frameshifting by stable downstream and upstream structures, respectively.

### 2.7. Ribosomal Flanking mRNA Structures also Modulate Bidirectional Frameshifting When an A-site Occupying Codon Becomes a Hungry Codon

Frameshifted translation products have been reported in research on protein overexpression in *E. coli.* [28]. Such hungry codon-mediated frameshifting has also been proposed to be responsible for observed −1 frameshifting of Huntingtin in Huntington’s disease due to the extensive use of glutamine CAG codon [29]. In this case, appropriate A-site codon occupation could be delayed due to low concentrations of cognate charged tRNA, leading to ribosome pausing. This is similar to an A-site sitting stop codon without its cognate release factor RF2 as described above. To see if the flanking structure effects are also applicable in hungry codon-mediated frameshifting regulation, we used a cysteine codon (UGU) to replace the UGA stop codon in the ribosomal A-site (Figure 8A). The cysteine concentration in the in vitro translation system was then reduced to mimic the hungry codon condition. Because GST-Rluc reporter possesses 4 cysteine amino acid residues upstream of the CUUUGA site, sequential dilutions of cysteine concentration were performed to optimize experimental conditions to ensure detectable levels of translation products while keeping the cysteine concentration as low as possible (Figure S6). By generating UGU (Cys) as a hungry codon at the ribosomal A-site, we observed +1 and −1 frameshifting stimulation by upstream H1 hairpin and downstream mPK, respectively (lanes 2 and 3 in Figure 8C). By contrast, destabilizing both structures by Watson-Crick base-pairing disruption (9G10A of H1 and G34C of mPK) almost abolished −1 frameshifting activity and strongly reduced +1 frameshifting activity (lane 1), while co-existence of stable flanking structures led to the compromise of both frameshifting pathways (lane 4). Thus, delay or interference in ribosomal A-site occupancy represents a parameter for translational reading-frame switch regulation that can be further tuned bi-directionally via stable mRNA duplexes flanking the elongation ribosome (Figure 8D).

## 3. Discussion

### 3.1. Upstream Hairpin Juxtaposing E-Site as the Functional Mimicry of Internal SD Mediated Duplex

The +1 stimulation and −1 attenuation activities of a co-translationally refolding RNA hairpin in 70S translation system suggest that it is a functional mimicry of an internal SD-mediated duplex that forms between mRNA and rRNA. Therefore, their frameshifting regulation properties may share similar mechanisms. Extensive mutagenesis of E- site codon composition has demonstrated that E-site codon-anticodon stability reversely correlates with +1 frameshifting efficiency in CUUUGA frameshifting sites [30,31] and was proposed to be related to the E-site tRNA dissociation. Consistently, biochemical experiments on RF2 +1 frameshifting have shown that extending the internal SD-mediated duplex by base-pairing with E- site sequences can enhance +1 frameshifting efficiency and accelerate the dissociation of E-site tRNA [24]. Alternatively, the involvement of peptidyl-tRNA dissociation has also been proposed based on toe-printing analysis [32]. By contrast, the mPK −1 PRF stimulator downstream of a non-XXXYYYZ sequence was shown to delay E-site tRNA dissociation [33]. Thus, it is tempting to explain the reverse role in frameshifting regulation by the structures flanking different frameshifting sites through the mediation of opposite effects in the dissociation of E-site tRNA. However, our experimental approach was not capable of kinetic measurement for E-site tRNA dissociation although limited mutagenesis analysis of E-site sequence composition in Figure 5 suggests that stability of codon-anticodon interaction is not the main cause for switch in frameshifting pathways. Alternatively, upstream duplexes juxtaposing the E-site could simply act as a roadblock to hinder the backward movement of ribosome to attenuate −1 PRF. Nevertheless, our finding of co-existence of +1 and −1 frameshifting under the same sequence background represents an ideal framework for studying frameshifting pathway regulation by E-site tRNA dissociation kinetics as well as flanking structures via single-molecule approaches in the future.

### 3.2. Mechanisms of the Non-Canonical −1 Frameshifting in +1 Frameshift-Prone CUUUGA Site

Kinetic modeling and experimental analysis have suggested that −1 PRF can occur in either accommodation or two sequential post-translocation steps along the X XXY YYZ slippery site [34]. However, the non-canonical −1 frameshifting observed in sequence CCUUUGA does not fit into the XXXYYYZ consensus for −1 PRF slippery sequence. In a recent model of UUUAAAG-mediated −1 PRF, mPK was proposed to prolong elongation factor G (EF-G) release during post-translocation stage to interfere with the swiveling of the 30S subunit head and the coupled translocation [35,36,37,38], allowing the ribosome to sample alternative reading-frames [14]. By contrast, single-molecule fluorescence analysis of the DNAX −1 PRF motif argued that the accommodation step during delayed EF-G release determines reading-frame selection [13]. As a downstream mPK and the absence of RF2 are both required for the non-canonical −1 frameshifting in CUUUGA site, together with the results in Figure 6B, they support a model of UGA codon occupying the A-site followed by a 5-nucleotide spaced downstream mPK upon frameshifting (Figure 6C). Since EF-G has probably dissociated from the ribosome after the translocation step that positions the UGA codon into the A-site, it seems probable that reverse of 30S head swiveling is completed [38]. However, this raises questions about the role of the downstream hairpin or mPK in this process. Alternatively, downstream structures could still delay the translocation process to allow prolonged reading-frame sampling by the ribosome [13,14,39]. During this process the 0 frame and −1 frame codons in the A-site can both compete for their cognate ligands with the −1 frame pathway prevailing in the absence of RF2. Furthermore, slippage of peptidyl-tRNA by one nucleotide in the −1 frame is facilitated by an identical nucleotide bridging the 0 frame E- and P-sites as revealed in Figure 5, eventually leading to repositioning of the −1 frame codons in the P- and A- sites. However, our data are incapable of distinguishing the order between −1 frame slippage of peptidyl-tRNA and −1 frame A-site tRNA accommodation (pathways A and B in Figure 6C). Within these contexts, the finding that the duplex structures flanking the two sides of ribosomal mRNA-binding channel play reverse roles in frameshifting regulation complements current proposed −1 PRF stimulation models of downstream structures for a more complete picture of the dynamic interplay between ribosome and mRNA in frameshifting regulation.

### 3.3. Stable mRNA Structure Unwinding and Refolding in CAG Trinucleotide Repeat Expansion

This study has demonstrated that −1 PRF attenuation by a co-translationally refolding upstream hairpin is conserved between 70 S and 80S ribosomes. It further suggests that frameshifting attenuation activity is an intrinsic property of duplex-mediate structures that flank the mRNA-binding channel of an elongating ribosome, and could be masked by the composition of the sequence occupied by the ribosome. Furthermore, the tendency of an A-site empty ribosome to shift its reading-frame in either direction depending on the existence of flanking structures could provide a working model for unusual frameshifting events mediated by hungry codons in both 70S and 80S ribosomes. Unusual −1 frameshifting without an XXXYYYZ shifty site has been reported for the CAG trinucleotide expansion region of the MJD-1 transcript in spinocerebellar ataxias 3 as well as huntingtin in Huntington’s disease [29,40]. Experimental evidence from CAG repeat expansion in huntingtin suggested that the consumption of glutamine-charged tRNA by the expanded repeats makes the glutamine-decoding CAG a hungry codon and eventually makes it frameshift-prone in the presence of CAG-mediated hairpin structures [41]. However, repeated CAG sequences lack an identical nucleotide to bridge the 0 frame E- and P- sites, and is consistent with the weak −1 frameshifting efficiency [29]. Additionally, long repeats are capable of generating both upstream and downstream CAG-mediated hairpins to flank the ribosome to further compromise frameshifting efficiency via frameshifting attenuation effects. By contrast, +1 frameshifting has also been reported for CAG repeat in huntingtin with the involvement of a weak UUCC +1 shifty site upstream of the expanded CAG repeats [42]. As expanded CAG repeats can form a downstream stable hairpin while an upstream CAG mediated hairpin refolds, attenuation of +1 frameshifting by a downstream hairpin could thus mask this mechanism.

## 4. Materials and Methods

### 4.1. Plasmids, Reporter Design and Construction

A pET GST-Rluc reporter was designed as the backbone for cloning of different frameshifting signals to examine the frameshifting activities using radioactivity-based frameshifting assay in vitro. To construct pET GST-Rluc, *Renilla* luciferase gene (Rluc) was amplified using p2luc vector [22] as the template, and then cloned into *EcoRI*/*NotI* sites of pGEX-4T-1 (GE Healthcare), generating a GST-Rluc fusion protein gene (GST-Rluc). The gene of GST-Rluc was then amplified and inserted into *NheI*/*NotI* sites of pET-21a (+) (Merck Co. Inc.) to form pET GST-Rluc. To facilitate non-shifted and shifted translation products separation by electrophoresis, translation termination sites were designed into each reading-frame with optimal spacing among these stop codons. The stop codon in the 0 frame was provided by the individual frameshifting elements under examination. Unless specified, the out of frame premature stop codons were designed into the N-terminal region of Rluc ORF of the pET GST-Rluc reporter. Due to the existence of multiple +1 and −1 frame stop codons in the N-terminal region of Rluc ORF, further deletions and site-directed point mutations were performed to generate pET GST-Rluc-1 for tracking single frameshifting event (−1 or +1) and double frameshifting events (−1 and +1). The nucleotide sequences of N-terminal regions of Rluc ORF in pET GST-Rluc-1 are shown below with the two boldly typed TAA corresponding to premature out of frame stop codons. 5′-A**TAA**CTTCGAAAGTTTATGATCCAGAACAAAGGA AACGGAAACTGGTCCGCAGTGGTGGGCCAGATGTACACAAA**TAA**-3′. (Notice: In single frameshifting events, the second boldly typed TAA was used as the termination site of −1 or +1 frameshifting (Figure 2). In double frameshifting events (Figure 3, Figure 5, Figure 6 and Figure 7), the first boldly typed TAA was used as −1 frame stop codon, and the second boldly typed TAA was used as +1 frame stop codon.)

The pET GST-FLAG-Rluc reporter was used to express FLAG-tagged translation products for immunoprecipitation. To generate GST-FLAG-Rluc, the pET GST-Rluc-1 reporter was used as the template. DNA oligo nucleotides R1 (5′-CTTGTCGTCATCGTCTTTGTAATCATCCGATTTTGGAGGATGGTC-3′) and R2 (5′-CGGGGATCCACGCGGAACCAGCTTGTCGTCATCGTCTTTGT-3′) were designed as reverse primers to contain nucleotides coding part of FLAG-tag (underlined) in the 5′ and 3′ regions, respectively. The 3′-part of R1 was designed to be complementary to the C-terminal end nucleotide sequences of GST while the 5′-part of R2 was extended with *BamHI* recognition sequences. By contrast, primer F-T7 (5′-GCGAAATTAATACGACTCACTATAGGG-3′) was designed as the forward primer to anneal with the sequence upstream of GST. Incubation of pET GST-Rluc-1 with R1 and F-T7 primers for 10 cycles of PCR amplification and followed by additional 20 cycles of PCR with the addition of F-T7 and R2 primers generated GST-FLAG with a flanked *NheI*/*BamHI* cutting sites. The amplified GST-FLAG containing cDNA was restriction enzymes treated and cloned into *NheI*/*BamHI* sites of the pET.GST-Rluc-1 reporter to replace the GST fragment to generate the GST-FLAG-Rluc reporter construct.

### 4.2. Recombinant DNAs and Mutagenesis

Unless specified, all frameshifting signals under examination were cloned into the pET GST-Rluc-1 reporter backbone for radioactivity-based frameshifting assay. The frameshifting elements containing different sequences of upstream and downstream structures flanking a slippery sequence were constructed by PCR-based ligation approach [43]. In brief, the elements were assembled by performing PCR using two to three pieces of chemically synthesized DNA oligo-nucleotides (from Genomics BioSci & Tech Corp at Taiwan) of partially overlapping sequences. Forward and reverse primers used for cDNAs amplification contain appropriately designed overlapping regions as well as *BamHI* and *EcoRI* restriction sites. The amplified cDNAs were then cloned into the *BamHI/EcoRI* sites of appropritate reporters. Following standard cloning procedures, the resultant recombinant vectors were transfromed into the DH5α strain of *Escherichia coli* and were selected by ampicillin. Site-directed mutagenesis in this study was constructed using the quick-change mutagenesis kit from Stratagene in accordance with the manufacturer’s instructions. All nuelcotide sequences of cloned and mutated frameshifitng elements were confirmed by DNA-sequencing analysis.

### 4.3. In Vitro Radioactivity-Based Frameshifting Assay

For in vitro frameshifting assay, nascent polypeptides were generated using the reconstituted 70S translation system (PURExpress ΔRF123 Kit, NEB) in accordance with manufacturer’s instructions. Unless specified, 200 ng mRNAs purified after transcription by T7 RNA polymerase (Thermo Fisher Scientific) was used as the templates in each 2.5 μL reaction, with RF2 excluded if required. To create cysteine deficiency condition for assaying hungry codon-mediated frameshifting, two different amino-acids mixture sets were added into the translation mixture (PURExpress Δ aa, tRNA Kit, NEB) separately. In each 2.5 uL reaction, 0.25 μL of 0.002 mM amino-acids mixture (NEB) and 0.25 μL of 1mM minus-Cysteine amino-acids mixture (Promega) were used. For each assay, 0.05 μL of 10 μCi/μL ^35^S labeled methionine (NEN) was added into the reaction, and incubated at 37 ℃ for 2 h. Samples were then resolved by 12% Sodium dodecylsulphate-polyacrylamide gel electrophoresis (SDS-PAGE), and exposed to a phosphorimager screen for quantification after drying of the gel. In ribonuclease-treated assay, specific amounts of ribonuclease A (Thermo Scientific, AM2274) were added into each reaction in the end. After incubation at room temperature for 15 mins, the reactions were quenched with 5 × SDS-gel loading buffer, and analyzed by SDS-PAGE. Frameshifting efficiencies were calculated, by dividing the counts of the shifted products by the sum of the counts for both shifted and non-shifted products, with calibration of the methionine content in each protein product.

### 4.4. Immunoprecipitation

A 20 μL cell-free translation reaction containing 200 ng -FLAG-CUUUGA-mPK reporter plasmid was incubated at 37 °C for 4 h. The reaction was then mixed with phosphate buffered saline (PBS) buffer (137 mM NaCl, 2.7 mM KCl, 10 mM Na_2_HPO_4_, 1.8 mM KH_2_PO_4_, pH7.4) containing 1:100 dilution of anti-FLAG M2 (Sigma) in a final volume of 400 μL, and rotated overnight at 4 °C. The sample was then incubated with 160 μL Protein G Magnetic Sepharose Xtra (GE Healthcare) at 25 °C for 2 h with gentle shaking. Following incubation, the beads were washed three times with 320 μL PBS buffer, and boiled in 20 μL 5 × SDS-gel loading buffer at 95 °C for 5 minutes to elute FLAG-tagged polypeptides. The elution fractions were resolved by 10% SDS-PAGE, and then stained with Coomassie Brilliant Blue R-250 (PanReac). After the gel was destained with destaining buffer (20% methanol, 10% glacial acetate acid), the target band was sliced for mass spectrometry (MS) analysis.

### 4.5. In-Gel Digestion and Mass Spectrometry

The gel slice containing translation products excised from the resolved gels was cut into pieces for in-gel trypsin digestion. Briefly, the gel pieces were first washed with 25 mM triethylammonium bicarbonate buffer (TEABC) / 50% (*v/v*) acetonitrile (ACN). The washed samples were then reduced with 10 mM DTT at 65 °C for 1 h and alkylated with 55 mM iodoacetamide (IAM) at room temperature for another 1 h in the dark. Trypsin buffer (protein:trypsin = 40:1, *w/w*) was added to cover the gel pieces, and incubated at 37 °C for more than 16 h. Tryptic peptides were extracted by incubating with 5% formic acid (FA)/ 50% acetonitrile (ACN) for 15 min, and vacuum dried. The sample was dissolved in 12 μL 0.1% (*v/v*) formic acid (FA) and 4 μL used in LC-MS/MS analysis. The synthetic peptide standard was purchased from Kelowna International Scientific Inc. (Taiwan).

LC-MS/MS was performed using an Orbitrap Fusion Lumos Tribrid mass spectrometer (Thermo Fisher Scientific) equipped with a NanoSpray Flex ion source. The trypsin-treated sample was loaded onto an UltiMate 3000 nano LC system (C18 Acclaim PepMap NanoLC column, 25 cm × 75 μm I.D., 2 μm,100 Å) connected to a mass spectrometer. A segmented gradient of 90 min from 2% to 35% solvent B at a flow rate of 300 nL/min and a column temperature of 35 °C was used for separation with solvent A containing 0.1% formic acid in water and solvent B containing 100% acetonitrile with 0.1% formic acid. Mass spectrometry analysis was performed in data-dependent mode with Full-MS (externally calibrated to a mass accuracy of <5 ppm, and a resolution of 120,000 at *m/z* = 200) followed by HCD-MS/MS of the most intense ions in 3 s. High-energy collision activated dissociation (HCD)-MS/MS (resolution of 15,000) was used to fragment multiple charged ions within a 1.4 Da isolation window at a normalized collision energy of 32 eV. AGC target at 5e5 and 5e4 was set for MS and MS/MS analysis, respectively, with previously selected ions dynamically excluded for 180 s. Max. injection time was 50 ms. A customized database was used to search for the existence of translation peptides by matching the m/z value to the obtained MS spectra.

## Supplementary Materials

The following are available online at http://www.mdpi.com/1422-0067/19/12/3867/s1, Figure S1: t Radioactivity-based frameshifting analysis of a −1 PRF model system using GST-Rluc-1 reporter by in vitro translation in cell-free lysates of E. coli., Figure S2: The +1 frameshifting stimulated by duplex structures upstream of the CUUUGA frameshifting site are abolished in the presence of RF2., Figure S3: Potential RNA-protein adducts disappear upon RNase A treatment., Figure S4: Recovery of a potential −1 frame translation product for mass spectrometry analysis., Figure S5: RNase A treatment removes a low mobility band without affecting the three major translation products., Figure S6: Creating a cysteine hungry codon by reducing cysteine amino acid concentration in the in vitro translation system.
