# Supplementary material for: mRNA-Mediated Duplexes Play Dual Roles in the Regulation of Bidirectional Ribosomal Frameshifting
Source: Int J Mol Sci. 2018 Dec 4;19(12):3867. doi: 10.3390/ijms19123867 (PMC6321510; doi:10.3390/ijms19123867)
Supplement: Supplementary file 1 [file ijms-19-03867-s001.pdf]

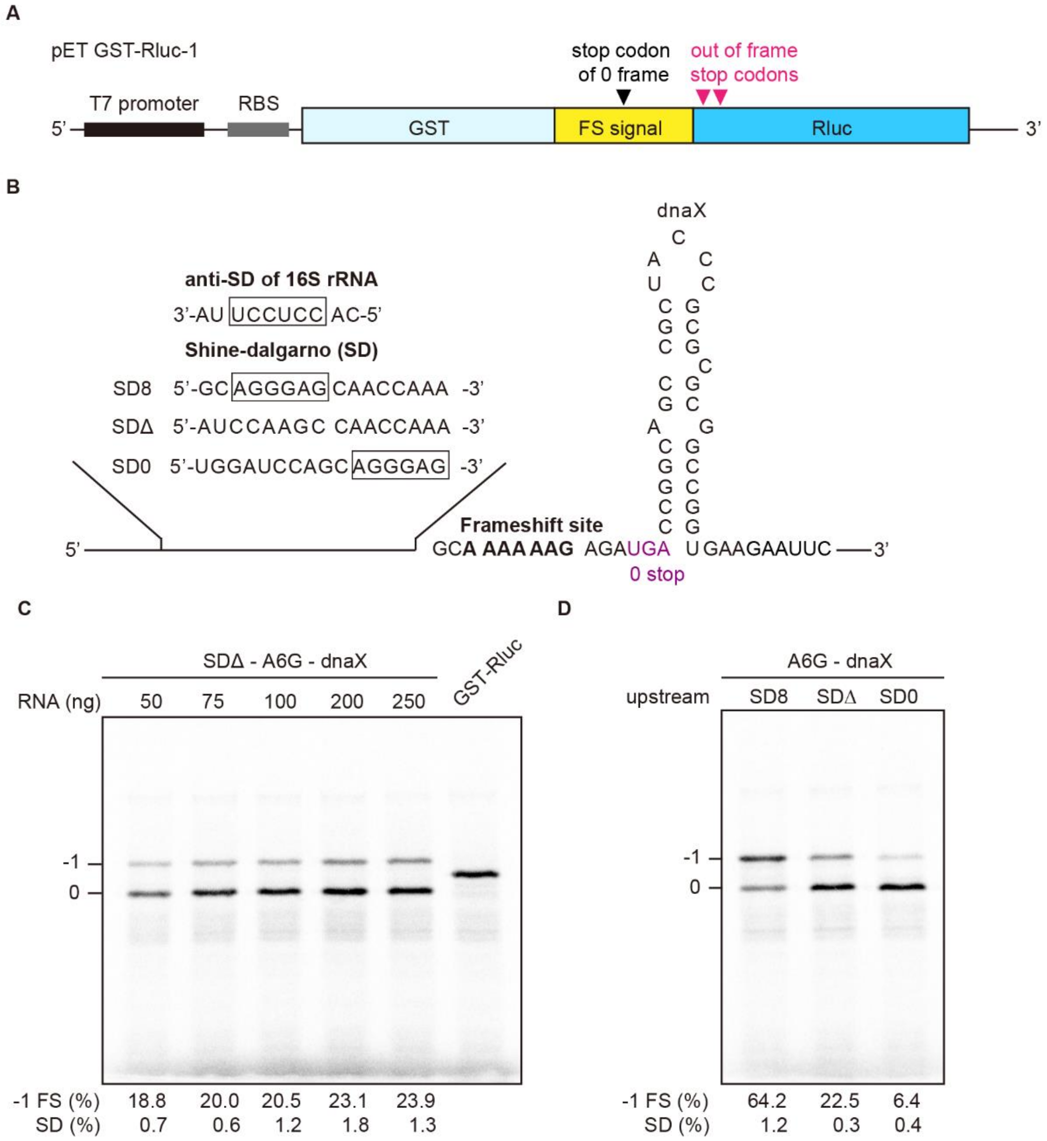

Figure S1. Radioactivity-based frameshifting analysis of a -1 PRF model system using GST-Rluc-1 reporter by *in vitro* translation in cell-free lysates of *E. coli.* (A) pET GST-Rluc-1 reporter designed for evaluating frameshifting efficiency by cloning of frameshifting signals into *BamHI* and *EcoRI* sites. The stop codons for three different reading-frames are annotated by reverse triangles. (B) A dnaX -1 PRF stimulation hairpin was placed downstream of an A6G slippery site and cloned into pET GST-Rluc-1 reporter to evaluate the effect of upstream internal SDs (boxed) of different spacing toward the frameshifting site. The UGA stop codons are used for 0 frame (in purple color) translation termination. (C) *In vitro* radioactivity based frameshifting assay to evaluate effects of different amounts of mRNA templates in dnaX hairpin stimulated -1 PRF efficiency. Calculated -1 PRF efficiencies are shown and the values displayed are means ± SD of three independent experiments. (D) *In vitro* radioactivity based frameshifting assays for constructs in (B) to evaluate the effects of different internal SDs in dnaX hairpin stimulated -1 PRF efficiency using 200 ng of mRNA for

each construct.

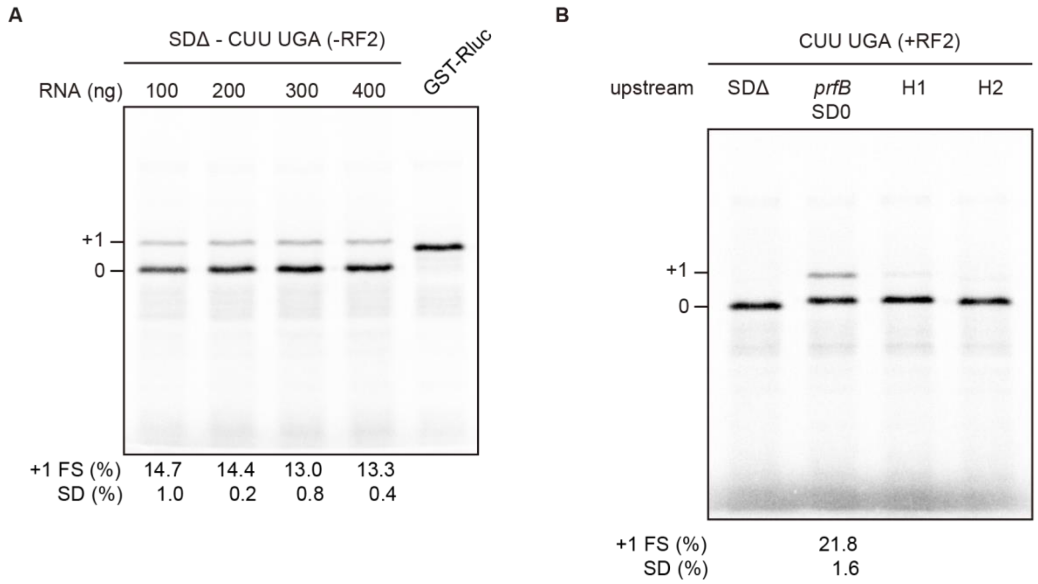

Figure S2. The +1 frameshifting stimulated by duplex structures upstream of the CUUUGA frameshifting site are abolished in the presence of RF2. (A) *In vitro* radioactivity based frameshifting assay to evaluate effect of different amounts of mRNA templates in CUUUGA frameshifting site mediated +1 PRF efficiency. Calculated +1 PRF efficiencies are shown and the values displayed are means ± SD of three independent experiments. (B) *In vitro* radioactivity based +1 PRF assays were performed and analyzed with the same conditions as those described in Figure 2D except that RF2 was added in the reaction.

A

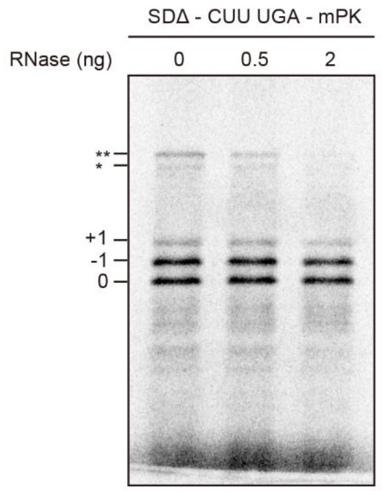

Figure S3. Potential RNA-protein adducts disappear upon RNase A treatment. SDS-PAGE analysis of ^{35}S methionine-labeled *in vitro* translation products with addition of different amounts of RNase before gel-loading. The potential RNA-protein adducts are annotated by asterisks.

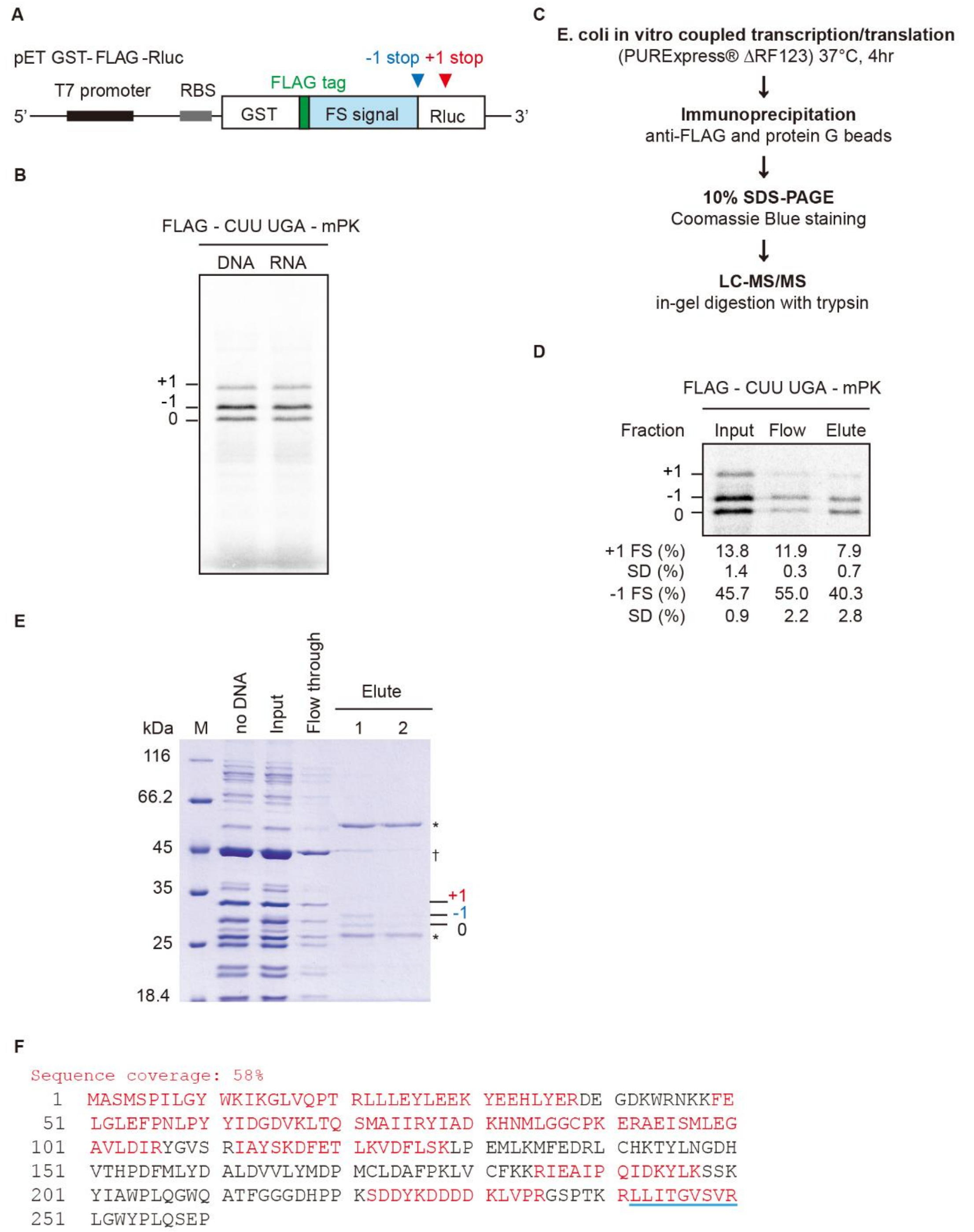

Figure S4. Recovery of a potential -1 frame translation product for mass spectrometry analysis. (A) Schematic drawing of construct (FLAG-CUUUGA-mPK) designed for frameshifting products recovery with a FLAG-tag. It was obtained by cloning frameshifting signals containing CGCCUUUGA and a downstream mPK into the *BamHI*/*EcoRI* sites of pET GST-FLAG-Rluc reporter. (B) SDS-PAGE analysis of frameshifting patterns of ^{35}S methionine-labeled *in vitro* translation reactions using purified mRNA or transcription-coupled translation (DNA). (C) Scheme for frameshifting products recovery. (D) SDS-PAGE analysis of ^{35}S methionine-labeled

translation products expressed from construct in (A) using the recovery scheme in (C). The analytical scale *in vitro* translation was tracked by ^{35}S-labeled methionine. (E) Coomassie Blue stained SDS-PAGE of the recovered translation products from transcription-coupled translation in cell-free *E. coli.* lysates using construct in (A). Asterisks indicate bands corresponding to the heavy/light chains of anti-FLAG M2 antibody used during immunoprecipitation of the lysates. The cross symbol indicates a band that is probably the result of cross-reaction with a strong protein product in the lysates. (F) Complete amino acid sequences of the predicted fusion protein with the sequences of peptides identified by LC MS/MS typed in red. The amino acid sequence for the peptide identified to span 0 and -1 frames is underlined in blue color. A 10 amino-acids peptide (RLLITGVSVR) spanning 0 and -1 frames was also identified and was probably the result of mis-cleavage in trypsin treatment.

A

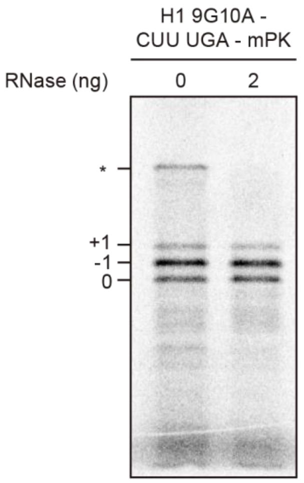

Figure S5. RNase A treatment removes a low mobility band without affecting the three major translation products. SDS-PAGE analysis of ^{35}S methionine-labeled *in vitro* translation products with addition of different amounts of RNase A before gel-loading. The potential RNA-protein adducts are annotated by an asterisk.

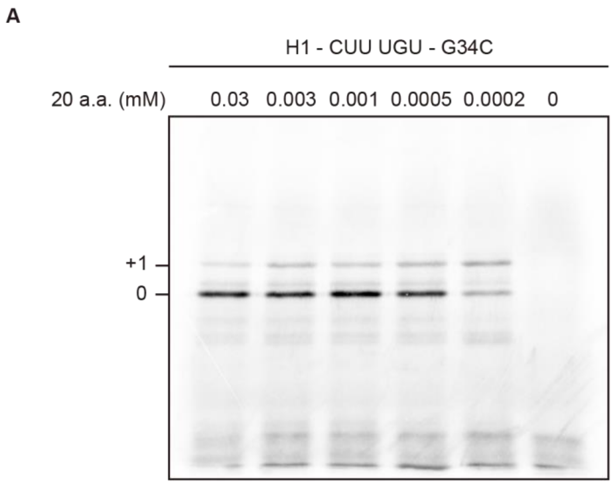

Figure S6. Creating a cysteine hungry codon by reducing cysteine amino acid concentration in the *in vitro* translation system. A minus-cysteine amino-acids mixture (-Cys) was combined with an amino-acids mixture containing all 20 amino acids of different dilution factors for use in *in vitro* translation to screen for optimal condition suitable for hungry codon study. Without the addition of 20 amino-acids mixture (lane 6), the translation of full-length product was blocked due to the removal of cysteine in the minus-cysteine amino-acids mixture, whereas the addition of 20 amino-acids mixture ensured full-length product translation (0-frame). The +1 frame product became dominant when 0.0002 mM combination was used as the experimental condition.
